# Coherent ac spin current transmission across an antiferromagnetic CoO insulator

**DOI:** 10.1038/s41467-019-13280-5

**Published:** 2019-11-20

**Authors:** Q. Li, M. Yang, C. Klewe, P. Shafer, A. T. N’Diaye, D. Hou, T. Y. Wang, N. Gao, E. Saitoh, C. Hwang, R. J. Hicken, J. Li, E. Arenholz, Z. Q. Qiu

**Affiliations:** 1 Department of Physics, University of California at Berkeley, Berkeley, CA 94720 USA; 2 Advanced Light Source, Lawrence Berkeley National Laboratory, Berkeley, CA 94720 USA; 3 WPI Advanced Institute for Materials Research, Tohoku University, Sendai, 980-8577 Japan; 4 Korea Research Institute of Standards and Science, Yuseong, Daejeon, 305-340 Korea; 5 Department of Physics and Astronomy, University of Exeter, Stocker Road, Exeter, Devon, EX4 4QL UK; 6 International Center for Quantum Materials, School of Physics, Peking University, Beijing, 100871 China

**Keywords:** Materials science, Nanoscience and technology, Physics

## Abstract

The recent discovery of spin current transmission through antiferromagnetic insulating materials opens up vast opportunities for fundamental physics and spintronics applications. The question currently surrounding this topic is: whether and how could THz antiferromagnetic magnons mediate a GHz spin current? This mismatch of frequencies becomes particularly critical for the case of coherent ac spin current, raising the fundamental question of whether a GHz ac spin current can ever keep its coherence inside an antiferromagnetic insulator and so drive the spin precession of another ferromagnet layer coherently? Utilizing element- and time-resolved x-ray pump-probe measurements on Py/Ag/CoO/Ag/Fe_75_Co_25_/MgO(001) heterostructures, here we demonstrate that a coherent GHz ac spin current pumped by the Py ferromagnetic resonance can transmit coherently across an antiferromagnetic CoO insulating layer to drive a coherent spin precession of the Fe_75_Co_25_ layer. Further measurement results favor thermal magnons rather than evanescent spin waves as the mediator of the coherent ac spin current in CoO.

## Abstract (web summary)

The mechanism underpinning the frequency mismatch between THz magnons and the GHz spin currents observed in antiferromagnetic insulators remains unknown. Here, the authors demonstrate that, in a Py/Ag/CoO/Ag/Fe_75_Co_25_/MgO(001) heterostructure, a GHz spin current transmits coherently across the antiferromagnetic CoO insulating layer to drive a coherent spin precession of the ferromagnetic Fe_75_Co_25_ layer.

## Introduction

Antiferromagnetic (AFM) materials have emerged as promising candidates for spintronic technology^1–3^. In particular, the discovery of spin current transmission through AFM insulators^4–9^ promotes their potential use for local spin switching within magnetic devices^10^^,^^11^. It is believed that the spin current propagation in AFM insulators is governed by THz magnons^12^, which poses a great challenge for coherent GHz ac spin current injection e.g., by ferromagnetic resonance (FMR)^13^. Due to the absence of GHz magnons in most AFMs, coherent GHz spin currents in AFM insulators have only been discussed in terms of evanescent waves^14^ with other theoretical models^15–17^ averaging out the THz ac components to focus on the dc spin current. This model is adequate to describe incoherent spin current injection (e.g., spin Seebeck effect^6,8^), where only dc spin currents are observed. However, it raises fundamental questions for coherent ac spin current injection and transmission (e.g., by FMR), where the frequency range (~GHz) is significantly lower than typical AFM magnon frequencies (~THz). Although FMR damping measurements indicate the injection of a GHz coherent ac spin current into an AFM layer^4,18–21^, direct pump-probe measurements reveal that magnons in AFM insulators can carry net spins only in the THz range^22^. Therefore, it becomes critically important to answer the question whether or not a GHz coherent spin current can propagate coherently across an AFM insulator to drive a coherent spin precession of another FM layer. Here, we report on experimental investigations of spin pumping, propagation, and transmission of a coherent GHz spin current in Py/Ag/CoO/Ag/Fe_75_Co_25_/MgO(001) using element- and time-resolved X-ray Magnetic Circular Dichroism (XMCD) and X-ray Magnetic Linear Dichroism (XMLD)^23,24^.

## Results

### Sample preparation and characterization

Two samples of Py/Ag^(1)^/CoO/Ag^(2)^/Fe_75_Co_25_ were grown on top of MgO(001) substrates using Molecular Beam Epitaxy (Supplementary Fig. 1) with layer thicknesses of 30 nm Py (Ni_20_Fe_80_), 2.5 nm CoO, 5 nm Fe_75_Co_25_, and 2 nm Ag between Py and CoO for both samples. The 2 nm Ag^(1)^ between Py and CoO in these two samples permits a non-zero Py/CoO magnetic interlayer coupling, which is important for the CoO spin alignment and for the Py spin pumping into the CoO (Ref. ^14–17^). The Ag^(2)^ thickness (*d*_Ag_) between the CoO and the Fe_75_Co_25_ layers was varied from 2 nm to 10 nm in the two samples to control the CoO/Fe_75_Co_25_ magnetic interlayer coupling^25^, leading to the presence and absence of an equivalent interlayer coupling between the Py and Fe_75_Co_25_ magnetizations across the Ag/CoO/Ag spacer (Supplementary Fig. 3). For convenience, we will refer to these two samples as Py/Ag/CoO/Ag(2 nm)/Fe_75_Co_25_ and Py/Ag/CoO/Ag(10 nm)/Fe_75_Co_25_, respectively.

**Fig. 1:**
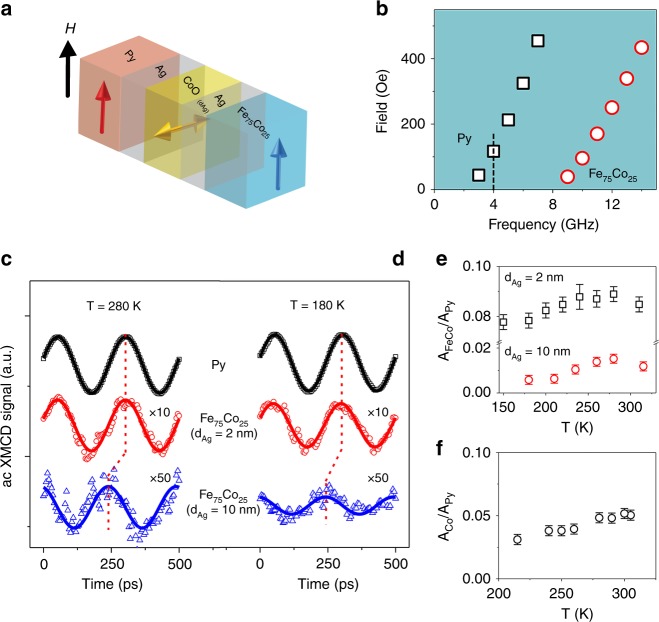
Ac spin current transmission through the CoO layer. **a** Schematic drawing of the spin configuration within the sample. **b** FMR fields for the Py and Fe_75_Co_25_ layers within the Py/Ag/CoO/Ag(10 nm)/Fe_75_Co_25_/MgO(001) sample. The dashed line shows where the ac XMCD/XMLD measurement was performed at the ALS. **c**, **d** Ac XMCD signals showing Py and Fe_75_Co_25_ spin precession at the Py FMR field at 280 K and 180 K, respectively. **e** Temperature-dependent ratio of Fe_75_Co_25_ precession amplitude to Py precession amplitude A_FeCo_⁄A_Py_ for the Py/Ag/CoO/Ag(2 nm)/Fe_75_Co_25_/MgO(001) and Py/Ag/CoO/Ag(10 nm)/Fe_75_Co_25_/MgO(001) samples. The error bars denote the standard errors of the means determined from the fittings of Fe_75_Co_25_ spin procession. **f** Temperature-dependent ratio of the Co precession amplitude to the Py precession amplitude for the Py/Ag/Co/CoO/MgO(001) sample

DC XMLD measurements reveal a perpendicular coupling between the Py spins and the CoO AFM spin axis^26^ (Fig. 1a) with a CoO Néel temperature of ~ 280 K (Supplementary Fig. 2). We first performed FMR measurements using conventional power absorption detection to characterize the FMR resonance fields. The results show distinct Py and Fe_75_Co_25_ resonance fields (Fig. 1b) with the Fe_75_Co_25_ FMR disappearing below 8 GHz. The distinctly different Py and Fe_75_Co_25_ resonance fields enable us to selectively excite the Py pump layer at 4 GHz and separately detect the spin current induced excitation of the Fe_75_Co_25_ sink layer (dashed line in Fig. 1b) using x-ray detected FMR (see Methods Section). In addition, hysteresis loop and FMR results confirm the presence and absence of Py/Fe_75_Co_25_ interlayer coupling across the Ag/CoO/Ag(2 nm) and Ag/CoO/Ag(10 nm) spacer layers, respectively (Supplementary Fig. 3). This allows us to separate the effect of the spin current from that of the interlayer coupling in driving the Fe_75_Co_25_ spin precession.

### Ac spin current transmission through the CoO layer

Using element- and time-resolved XMCD measurements, we measured the Py and Fe_75_Co_25_ spin precession at the Py FMR field for a 4 GHz rf excitation. At *T* = 280 K, the Co ac XMCD signal (Fig. 1c) clearly shows coherent Fe_75_Co_25_ spin precession in both Py/Ag/CoO/Ag(2 nm)/Fe_75_Co_25_ and Py/Ag/CoO/Ag(10 nm)/Fe_75_Co_25_ samples. Owing to the presence and absence of the interlayer coupling, the observed Fe_75_Co_25_ spin precession can be attributed to different mechanisms for the two samples. In Py/Ag/CoO/Ag(2 nm)/Fe_75_Co_25_ both the Py/Fe_75_Co_25_ interlayer coupling and the ac spin current contribute to the Fe_75_Co_25_ spin excitation, whereas it is dominated by the pure ac spin current in the Py/Ag/CoO/Ag(10 nm)/Fe_75_Co_25_ sample. This assertion is supported by the observation of different amplitudes and phase delays of the Fe_75_Co_25_ spin precession in the two samples. At 180 K, i.e., 100 K below the T_N_ of the CoO layer, we observe ~ 10% and ~ 60% decrease of the Fe_75_Co_25_ spin precession amplitude in Py/Ag/CoO/Ag(2 nm)/Fe_75_Co_25_ and Py/Ag/CoO/Ag(10 nm)/Fe_75_Co_25_ (Fig. 1c), respectively, confirming the presence of two different mechanisms driving the Fe_75_Co_25_ spin precession. To separate the interlayer coupling and the spin current contributions, we measured the temperature dependence of the Fe_75_Co_25_ precession amplitude normalized to the Py precession amplitude (*A*_FeCo_/*A*_Py_). In other words, the response of the spin sink layer is normalized to the strength of the spin source. The results (Fig. 1e) show that the *A*_FeCo_/*A*_Py_ values in both samples exhibit a broad peak around the CoO Néel temperature of 280 K, similar to the behavior observed for dc spin currents^6,8^. The difference in *A*_FeCo_/*A*_Py_ between these two samples is a temperature-independent constant, indicating that this difference is owing to the Py/Fe_75_Co_25_ interlayer coupling in Py/Ag/CoO/Ag(2 nm)/Fe_75_Co_25_ and that the common broad peak near 280 K is owing to transmission of a coherent ac spin current through the CoO layer. We then measured the Co spin precession in a Py(30 nm)/Ag(2 nm)/Co(1 nm)/CoO(2.5 nm)/MgO(001) reference sample (Fig. 1f) in which the Co(1 nm) serves as an indicator of the spin precession driven by the Py FMR before the ac spin current propagates through the CoO. The result shows a monotonic temperature dependence of the Co spin precession amplitude, suggesting that the broad peak in the Fe_75_Co_25_ spin precession amplitude near the CoO(2.5 nm) Néel temperature of 280 K is caused by the transmission of the ac spin current through the CoO. The temperature dependence of the Co spin precession amplitude in Fig. 1f is an interesting topic for future research, but is not the focus of the present work.

### Separation of spin current and interlayer coupling

**Fig. 2:**
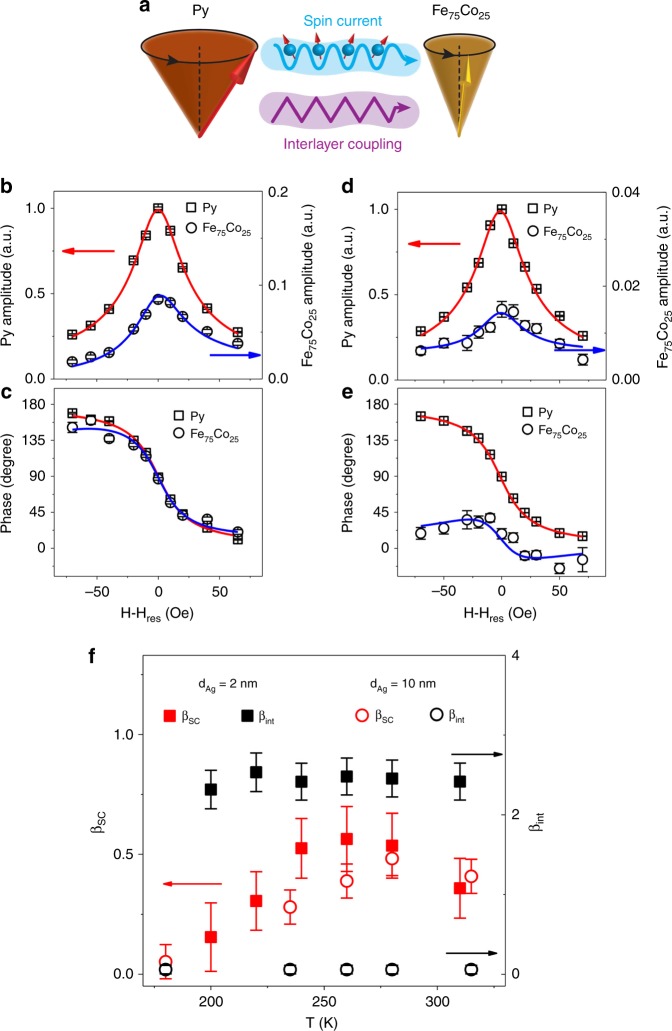
Separation of spin current and interlayer coupling contributions to the torque driving the Fe_75_Co_25_ spin precession. **a** Schematic drawing of ac spin current and interlayer coupling as the driving mechanisms of Fe_75_Co_25_ spin precession originating from the Py FMR. **b**, **d** Field dependence of the amplitude and **c**, **e** field dependence of the phase of Py and Fe_75_Co_25_ spin precession at 280 K from **b**, **c** Py/Ag/CoO/Ag(2 nm)/Fe_75_Co_25_ and **d**, **e** Py/Ag/CoO/Ag(10 nm)/Fe_75_Co_25_, respectively. Red and blue lines are fits to the Py and Fe_75_Co_25_ signals, respectively. The error bars denote the standard errors of the means determined from the fittings of FM spin procession. **f** Temperature dependence of the spin current (β_sc_) and interlayer coupling (β_int_) coefficients for the two samples. The error bars denote the standard errors of the means determined from the fittings of field-dependent FM spin processions based on Eq. (1) and Eq. (2)

Next, we measured the Py and Fe_75_Co_25_ spin precession for different magnetic fields above and below the Py FMR. Both the amplitude and phase of the Py and Fe_75_Co_25_ spin precession were extracted by fitting the ac XMCD phase delay scans with a sinusoidal function. The Py spin precession amplitude exhibits the Lorentzian shape $A_{{\mathrm{Py}}}^2\sim \Delta H^2/[(H - H_{{\mathrm{res}}})^2 + \Delta H^2]$ expected for FMR, whereas the phase varies as $tan\varphi _{{\mathrm{Py}}} = \Delta H/(H - H_{{\mathrm{res}}})$ (red lines in Fig. 2b–e), exhibiting a total phase shift of 180° as the field is swept through the resonance, where *H*_res_, Δ*H*, *A*_Py_,and *φ*_Py_ are the Py FMR field, FMR linewidth, spin precession amplitude, and phase of precession, respectively. The Fe_75_Co_25_ spin precession amplitude *A*_FeCo_ (Fig. 2b, d) exhibits a clear peak at the Py FMR field in both the Py/Ag/CoO/Ag(2 nm)/Fe_75_Co_25_ and Py/Ag/CoO/Ag(10 nm)/Fe_75_Co_25_ samples. As Fe_75_Co_25_ does not undergo FMR at 4 GHz, the peak in the Fe_75_Co_25_ precession amplitude at the Py FMR field proves that the enhanced Fe_75_Co_25_ spin precession must be induced by precession of the Py spins. To identify the different driving mechanisms in the two samples (Fig. 2a), we analyzed the Fe_75_Co_25_ precession phase *φ*_FeCo_. The Fe_75_Co_25_ precession phase in the Py/Ag/CoO/Ag(2 nm)/Fe_75_Co_25_ sample exhibits a monotonic field dependence, similar to that of the Py layer (Fig. 2c). This can be understood as being the result of the Py/Fe_75_Co_25_ interlayer coupling, which favors parallel alignment of the Fe_75_Co_25_ and Py spins (both dc and ac components). In contrast, the phase of the Fe_75_Co_25_ precession in the Py/Ag/CoO/Ag(10 nm)/Fe_75_Co_25_ sample (Fig. 2e) exhibits a clear bipolar behavior which is a fingerprint of spin current driven precession^21,23^.

To obtain a detailed quantitative understanding of the different mechanisms contributing to the Fe_75_Co_25_ precession, we consider the Landau–Lifshifts–Gilbert equation^21,23^, where the quantities driving the precession include the rf field (${\vec{\boldsymbol{h}}}_{{\mathrm{rf}}}$) from the coplanar waveguide (CPW), the effective Py/Fe_75_Co_25_ interlayer magnetic coupling energy ($- J_{{\mathrm{int}}}{\vec{\boldsymbol{m}}}_{{\mathrm{Py}}} \cdot {\vec{\boldsymbol{m}}}_{{\mathrm{FeCo}}}$), and an ac spin current generated by the Py FMR ($\alpha _{{\mathrm{Py}}}^{{\mathrm{sp}}}{\vec{\boldsymbol{m}}}_{{\mathrm{Py}}} \times \frac{{{\mathrm{d}}{\vec{\boldsymbol{m}}}_{{\mathrm{Py}}}}}{{{\mathrm{dt}}}}$), where $\alpha _{{\mathrm{Py}}}^{{\mathrm{sp}}}$ is the Py spin pumping coefficient, and ${\vec{\boldsymbol{m}}}_{{\mathrm{Py}}}$ and ${\vec{\boldsymbol{m}}}_{{\mathrm{FeCo}}}$ are the Py and Fe_75_Co_25_ magnetization unit vectors, respectively. The important difference between the interlayer coupling and the spin current mechanisms is that their associated driving torques have a 90° phase difference, resulting in a distinctly different phase behavior for the Fe_75_Co_25_ spin precession (see below). As compared with the Fe_75_Co_25_ spin precession amplitude ($A_{{\mathrm{FeCo}}}^0$) and phase ($\varphi _{{\mathrm{FeCo}}}^0$) driven by ${\vec{\boldsymbol{h}}}_{{\mathrm{rf}}}$ only, the modification of the Fe_75_Co_25_ spin precession amplitude and phase by the interlayer coupling and spin current can be described by (Supplementary Note 3)

1 $$\left| {\frac{{A_{{\mathrm{FeCo}}}}}{{A_{{\mathrm{FeCo}}}^0}}} \right| = \sqrt {1 + \left( {\beta _{{\mathrm{int}}}^2 + \beta _{{\mathrm{sc}}}^2} \right){\mathrm{sin}}^2\varphi _{{\mathrm{Py}}} + 2(\beta _{{\mathrm{int}}}{\mathrm{sin}}\varphi _{{\mathrm{Py}}}{\mathrm{cos}}\varphi _{{\mathrm{Py}}} + \beta _{{\mathrm{sc}}}{\mathrm{sin}}^2\varphi _{{\mathrm{Py}}})}$$

2 $${\mathrm{tan}}(\varphi _{{\mathrm{FeCo}}} - \varphi _{{\mathrm{FeCo}}}^0) = \frac{{\beta _{{\mathrm{int}}}{\mathrm{sin}}^2\varphi _{{\mathrm{Py}}} - \beta _{{\mathrm{sc}}}{\mathrm{sin}}\varphi _{{\mathrm{Py}}}{\mathrm{cos}}\varphi _{{\mathrm{Py}}}}}{{1 + \beta _{{\mathrm{int}}}{\mathrm{sin}}\varphi _{{\mathrm{Py}}}{\mathrm{cos}}\varphi _{{\mathrm{Py}}} + \beta _{{\mathrm{sc}}}{\mathrm{sin}}^2\varphi _{{\mathrm{Py}}}}}$$

where $\beta _{{\mathrm{int}}} = \frac{{M_{{\mathrm{Py}}}t_{{\mathrm{Py}}}}}{{M_{{\mathrm{FeCo}}}t_{{\mathrm{FeCo}}}}} \cdot \frac{{\gamma {\mathrm{J}}_{{\mathrm{int}}}}}{{\alpha _{{\mathrm{Py}}}\omega }}$ and $\beta _{{\mathrm{sc}}} = \frac{{M_{{\mathrm{Py}}}t_{{\mathrm{Py}}}}}{{M_{{\mathrm{FeCo}}}t_{{\mathrm{FeCo}}}}} \cdot \frac{{\alpha _{{\mathrm{Py}}}^{{\mathrm{sp}}}}}{{\alpha _{{\mathrm{Py}}}}}$ correspond to the interlayer coupling and spin current mechanisms, respectively. Equation (1) shows that both *β*_int_ and *β*_SC_ enhance the Fe_75_Co_25_ precession amplitude by generating a peak in *A*_FeCo_ in the vicinity of the Py resonance field, making it difficult to distinguish the spin current and the interlayer coupling effects purely by considering the precession amplitude. In contrast, Eq. (2) shows that only the spin current term (*β*_SC_) leads to a bipolar phase behavior in the vicinity of the Py FMR (*φ*_Py~90°_) with $\varphi _{{\mathrm{FeCo}}} > \varphi _{{\mathrm{FeCo}}}^0$ for H < *H*_Py,res0_ and $\varphi _{{\mathrm{FeCo}}} < \varphi _{{\mathrm{FeCo}}}^0$ for H > *H*_Py,res0_. With *φ*_Py_ derived from the Py data (red lines in Fig. 2c, e), we fit the Fe_75_Co_25_ spin precession amplitude and phase using Eqs. (1) and (2) with *β*_int_ and *β*_SC_ as fitting parameters. The results agree very well with the experimental data (blue lines in Fig. 2b–e). Figure 2f summarizes the *β*_int_ and *β*_SC_ values obtained for different temperatures. The interlayer coupling term *β*_int_ has a finite value in Py/Ag/CoO/Ag(2 nm)/Fe_75_Co_25_ but is virtually zero in Py/Ag/CoO/Ag(10 nm)/Fe_75_Co_25_. The spin current term *β*_SC_ exhibits similar values in both samples with a broad peak around the CoO Néel temperature. This result clearly shows the different contributions made by the interlayer coupling and the spin current to the Fe_75_Co_25_ spin precession in the two samples. Based on these observations, the enhancement of the Fe_75_Co_25_ spin precession around *T* = 280 K (Fig. 1e) can be attributed to an increase in the transmission of coherent ac spin current through the CoO layer around the Néel temperature. It is noteworthy that the equivalent Py/Fe_75_Co_25_ interlayer coupling across the Ag/CoO/Ag spacer shows relatively weak temperature dependence, even above the CoO Néel temperature. Although this is not the focus of the present work, we would like to mention that it is a well-known fact that the interlayer coupling across an AFM-insulating spacer exist well above T_N_^27,28^. The underlying mechanism has not been well understood and is still an ongoing research topic^29^.

### Probing the AFM CoO spin precession

**Fig. 3:**
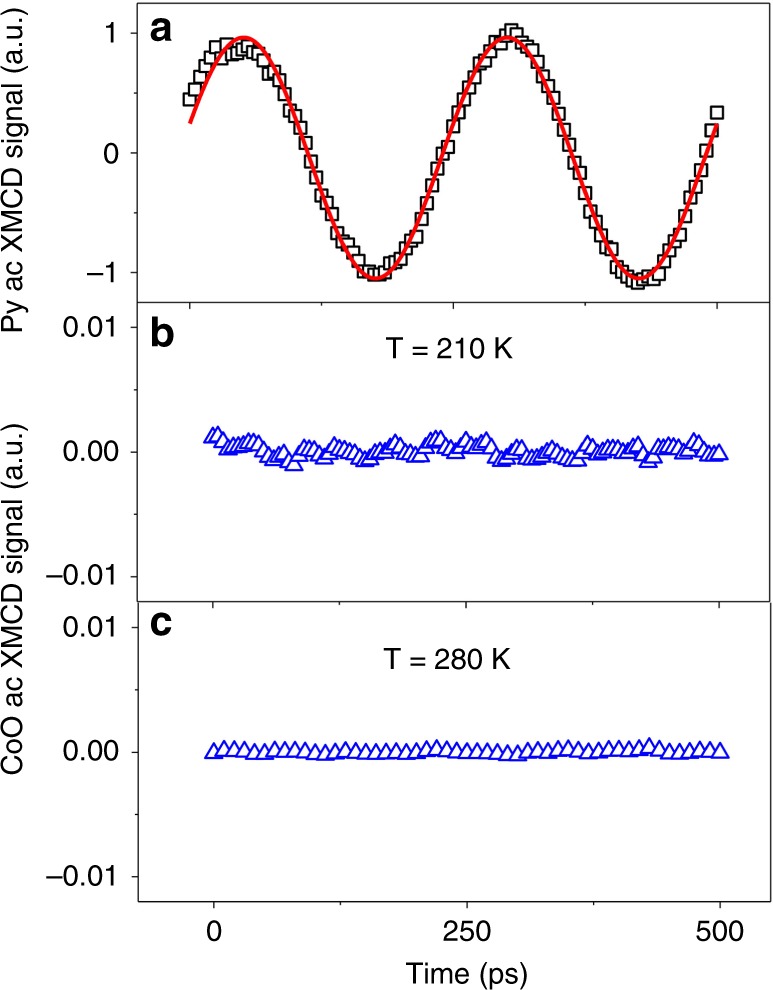
Measurement of AFM CoO spin axis precession. **a** Py ac XMCD signal at 210 K. CoO ac XMLD signal at **b** 210 K and **c** 280 K

To answer the question of whether the coherent ac spin current is carried by evanescent waves within the AFM^14^, which involves a coherent GHz precession of the AFM spin axis, we prepared a sample of Py(30 nm)/Ag(2 nm)/CoO(2.5 nm)/MgO(001) and performed ac XMLD measurements of the dynamics of the CoO moments. In detail, linearly polarized x-rays at normal incidence, with polarization axis tilted by 45° with respect to the CoO AFM spin axis, were utilized to detect the dynamic XMLD from the CoO layer. A rf evanescent GHz spin waves are excited within the CoO. However, our results show no detectable CoO ac XMLD signal both at 210 K and 280 K even with a data accumulation time much greater than that for the ac XMCD measurement (Fig. 3). Taking into account the noise level of the CoO ac XMLD signal, we estimate an approximate upper limit of the ac to dc signal ratio (ac XMLD divided by dc XMLD) to be < 0.00005 for the CoO precession at the Py FMR field. For comparison, the ac XMCD/dc XMCD ratio of Py and Fe_75_Co_25_ in Py/Ag/CoO/Ag(10 nm)/Fe_75_Co_25_ at the Py FMR field are estimated to be 0.011 and 0.00065, respectively. We would like to mention that the resonance frequency of CoO bulk modes is in THz range^30^, far above the 4 GHz frequency used in our experiment. Therefore, the absence of the CoO ac XMLD signal suggests the absence of CoO evanescent modes in our sample, but rather promotes the idea of spin current transmission mediated by thermal magnons.

## Discussion

It remains to be determined how THz thermal magnons can transport a coherent GHz spin current. It has been shown that a dc spin current is not carried by a single AFM thermal magnon mode. Instead, by lifting the population degeneracy of the left- and right-handed magnon modes of the AFM, i.e., by creating a right-handed magnon while annihilating a left-handed magnon of the same energy, a net spin angular momentum can be induced along the AFM spin axis without altering the energy state of the system considerably^12^. If GHz precession is considered to be an adiabatic process for the THz AFM magnons, then it may be possible for the THz AFM magnons to carry a coherent GHz spin current. Here, the instantaneous ac spin current would be transmitted as if it were a dc spin current, provided that the instantaneous spin orientation has a finite component parallel to the spin axis of the THz magnons. We speculate this condition is more likely to occur in a more isotropic AFM insulator (e.g., CoO and NiO) near the Néel temperature. However, for AFM insulators with a strong uniaxial anisotropy, where the spin axes of all magnons lie along the same direction, we would expect a spin current with spin orientation perpendicular to the AFM spin axis to be filtered out^8^. This picture is consistent with our observation that the ac spin current transmission in CoO behaves in a similar way to the dc spin current transmission, with an enhancement around the Néel temperature. This speculation or interpretation remains as an open question for future studies. Obviously, more theoretical work is needed to explore these mechanisms and to address this issue quantitatively.

In summary, we have measured spin precession using element- and time-resolved XMCD and XMLD in Py/Ag/CoO/Ag/Fe_75_Co_25_/MgO(001), using precessional pumping of the Py to generate a coherent GHz ac spin current. We find that the Fe_75_Co_25_ spins can be driven coherently through the AFM CoO by the GHz ac spin current with a peak in the precession amplitude around the CoO Néel temperature. In contrast, no GHz ac XMLD signal was observed from the CoO, suggesting that transmission of the spin current through the AFM CoO is not mediated by evanescent GHz frequency waves.

## Methods

### X-ray pump-probe measurements

X-ray pump-probe measurements were performed at Beamline 4.0.2 of the Advanced Light Source (ALS) at Lawrence Berkeley National Laboratory (LBNL). Microwave current of 4 GHz frequency is delivered to a CPW that generates a rf field at the sample. The rf excitation is synchronized to the ~ 500 MHz electron bunch frequency of the storage ring, to ensure a fixed phase relation between the microwave pump signal and the probing x-ray pulses. This enables a stroboscopic measurement of the excited magnetic moments. We carried out phase delay scans by incrementally changing the time delay of the rf field with respect to the x-ray probe pulses, enabling us to map out the magnetization precession and to obtain detailed information about the precession amplitude and phase. The CPW contains a small hole (diameter ~ 0.5 mm) in the signal line, which allows transmission of x-rays to the sample without affecting the CPW performance. A photodiode collects the luminescence yield from the sample to obtain the XMCD/XMLD signal. The x-ray incidence angle was 50° relative to the surface normal of the sample so that the in-plane component of the spin precession excited by the CPW could be obtained by element-resolved XMCD measurements as a function of the time delay of the microwave rf field^23,24^. For the XMCD measurements, the x-ray energy was tuned to the Ni *L*_3_ edge (852.5 eV) and the Co *L*_3_ edge (778.2 eV) to observe the dynamic Py and Fe_75_Co_25_ XMCD signals, respectively. For the linear dichroism measurements, the photon energy was tuned to 778.8 eV, to obtain the maximum CoO XMLD effect.

## Supplementary information

Supplementary Information

## Data Availability

Data are available from the corresponding author upon reasonable request.

## References

[1] Jungwirth T, Marti X, Wadley P, Wunderlich J (2016). Antiferromagnetic spintronics. Nat. Nanotech..

[2] Gomonay O, Baltz V, Brataas A, Tserkovnyak Y (2018). Antiferromagnetic spin textures and dynamics. Nat. Phys..

[3] Baltz V (2018). Antiferromagnetic spintronics. Rev. Mod. Phys..

[4] Wang H, Du C, Hammel PC, Yang F (2014). Antiferromagnonic spin transport from Y_3_Fe_5_O_12_ into NiO. Phys. Rev. Lett..

[5] Hahn C (2014). Conduction of spin currents through insulating antiferromagnetic oxides. Europhys. Lett..

[6] Lin W, Chen K, Zhang S, Chien CL (2016). Enhancement of thermally injected spin current through an antiferromagnetic insulator. Phys. Rev. Lett..

[7] Qiu Z (2016). Spin-current probe for phase transition in an insulator. Nat. Commun..

[8] Qiu Z (2018). Spin colossal magnetoresistance in an antiferromagnetic insulator. Nat. Mater..

[9] Lebrun R (2018). Tunable long-distance spin transport in a crystalline antiferromagnetic iron oxide. Nature.

[10] Wadley P (2016). Electrical switching of an antiferromagnet. Science.

[11] Oh Y-W (2016). Field-free switching of perpendicular magnetization through spin–orbit torque in antiferromagnet/ferromagnet/oxide structures. Nat. Nanotech..

[12] Cheng R, Xiao J, Niu Q, Brataas A (2014). Spin pumping and spin-transfer torques in antiferromagnets. Phys. Rev. Lett..

[13] Jiao H, Bauer GEW (2013). Spin backflow and ac voltage generation by spin pumping and the inverse spin hall effect. Phys. Rev. Lett..

[14] Khymyn R, Lisenkov I, Tiberkevich VS, Slavin AN, Ivanov BA (2016). Transformation of spin current by antiferromagnetic insulators. Phys. Rev. B.

[15] Rezende SM, Rodríguez-Suárez RL, Azevedo A (2016). Diffusive magnonic spin transport in antiferromagnetic insulators. Phys. Rev. B.

[16] Chen K, Lin W, Chien CL, Zhang S (2016). Temperature dependence of angular momentum transport across interfaces. Phys. Rev. B.

[17] Takei S, Moriyama T, Ono T, Tserkovnyak Y (2015). Antiferromagnet-mediated spin transfer between a metal and a ferromagnet. Phys. Rev. B.

[18] Frangou L (2016). Enhanced spin pumping efficiency in antiferromagnetic IrMn thin films around the magnetic phase transition. Phys. Rev. Lett..

[19] Merodio P (2014). Direct observation of exchange bias related uncompensated and absorption mechanisms of spin currents in Ir_20_Mn_80_ and Fe_50_Mn_50_ polycrystalline films by ferromagnetic resonance and spin pumping. Appl. Phys. Lett..

[20] Moriyama T (2017). Magnetic moment orientation-dependent spin dissipation in antiferromagnets. Phys. Rev. Lett..

[21] Baker AA (2016). Anisotropic absorption of pure spin currents. Phys. Rev. Lett..

[22] Kampfrath T (2011). Coherent terahertz control of antiferromagnetic spin waves. Nat. Photon..

[23] Li J (2016). Direct detection of pure ac spin current by x-ray pump-probe measurements. Phys. Rev. Lett..

[24] Marcham MK (2013). Phase resolved x-ray ferromagnetic resonance measurements of spin pumping in spin valve structures. Phys. Rev. B.

[25] Meng Y (2012). Magnetic interlayer coupling between antiferromagnetic CoO and ferromagnetic Fe across a Ag spacer layer in epitaxially grown CoO/Ag/Fe/Ag(001). Phys. Rev. B.

[26] Wu J (2010). Direct measurement of rotatable and frozen CoO spins in exchange bias system of CoO/Fe/Ag(001). Phys. Rev. Lett..

[27] Liu ZY, Adenwalla. S (2003). Oscillatory interlayer exchange coupling and its temperature dependence in (Pt/Co)_3_/NiO/(Co/Pt)_3_ multilayers with perpendicular anisotropy. Phys. Rev. Lett..

[28] Baruth A (2006). Origin of the interlayer exchange coupling in (Co/Pt)/NiO/(Co/Pt) multilayers studied with XAS, XMCD, and micromagnetic modeling. Phys. Rev. B.

[29] Cheng R, Xiao D, Zhu JG (2018). Interlayer couplings mediated by antiferromagnetic magnons. Phys. Rev. Lett..

[30] Satoh T (2017). Excitation of coupled spin–orbit dynamics in cobalt oxide by femtosecond laser pulses. Nat. Commun..

