# Supplementary material for: Coherent ac spin current transmission across an antiferromagnetic CoO insulator
Source: Nat Commun. 2019 Nov 20;10:5265. doi: 10.1038/s41467-019-13280-5 (PMC6868243; doi:10.1038/s41467-019-13280-5)
Supplement: Supplementary file 1 — Supplementary Information [file 41467_2019_13280_MOESM1_ESM.pdf]

Supplementary file:

Coherent ac spin current transmission across an antiferromagnetic CoO insulator

Li *et al.*

Supplementary Note 1. Sample growth

Epitaxial Py/Ag/CoO/Ag/$Fe_{75}Co_{25}$/MgO(001) and Py/Ag/CoO/MgO(001) samples were grown by molecular beam epitaxy in an ultrahigh-vacuum system with a base pressure of $5x10^{-10}$ Torr. All films were grown at room temperature. $Fe_{75}Co_{25}$ was grown by evaporation of Fe and Co in a 3:1 ratio. The CoO film was grown by evaporating Co from an e-beam target at an oxygen atmosphere of $2.0x10^{-6}$ Torr. Ag and Py were grown from thermal crucibles. Low-energy electron diffraction (LEED) results show a single-crystalline bcc structure for $Fe_{75}Co_{25}$ and an fcc structure for the CoO, the NiO, and the Ag layer with the $Fe_{75}Co_{25}$ [100] axis parallel to the CoO[110] and Ag[110] axes. After the growth of a polycrystalline Py layer, the samples were capped with a 3 nm MgO protective layer.

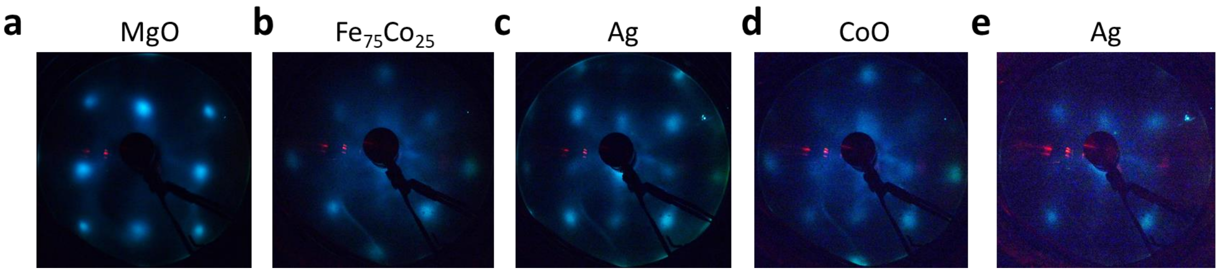

Supplementary Figure 1| Epitaxial growth of the multilayer sample. LEED pattern of each layer from the sample confirms good single-crystalline $Fe_{75}Co_{25}$, Ag, CoO, and Ag layers in Py/Ag/CoO/Ag/$Fe_{75}Co_{25}$/MgO(001).

Supplementary Note 2. XMLD measurements

XMLD measurements were performed at the Co L_3 edge by changing the linear polarization angle (ϕ) of the x-rays relative to the CoO[110] axis of Py(30 nm)/Ag(2 nm)/CoO(2.5 nm)/MgO(001). Supplementary Figure 2**b**,**c** show CoO spectra at x-ray polarizations of $\phi = 0°$ and $\phi = 90°$ for a magnetic field applied along the y and z axes. Recalling that the XMLD effect is revealed by the difference between the $\phi = 90°$ and $\phi = 0°$ spectra, observation of opposite CoO XMLD effects for the magnetic field applied along the y and z axes shows that the CoO AFM spins are coupled and rotatable with the Py spins. Supplementary Figure 2**d** shows the CoO L_3 ratio R_{L3} (which is defined as the ratio of intensity at a photon energy of 778.2 eV to that at 778.7 eV) as a function of x-ray polarization angle. The CoO R_{L3} exhibits the expected $cos^2\phi$ dependence with a maximum value at $\phi = 90°$ for field applied along the y axis and at $\phi = 0°$ for field applied along the z axis, respectively. Based on the rule that the CoO R_{L3} under these conditions should reach its maximum value for x-ray polarization parallel to the CoO spin axis [1], we conclude that the AFM CoO spins in Py(30 nm)/Ag(2 nm)/CoO(2.5 nm)/MgO(001) are coupled to and rotatable with the Py spins, and that the CoO AFM spin axis is perpendicular to the Py spin axis in the film plane. The CoO L_3 ratio difference ΔR_{L3}, defined as $\Delta R_{L3} \equiv |R_{L3}(\phi = 0°) - R_{L3}(\phi = 90°)|$,

decreases at higher temperatures and reaches zero at around 280 K [Supplementary Figure 2**e**], indicating that the Néel temperature is around 280 K for a 2.5 nm CoO layer.

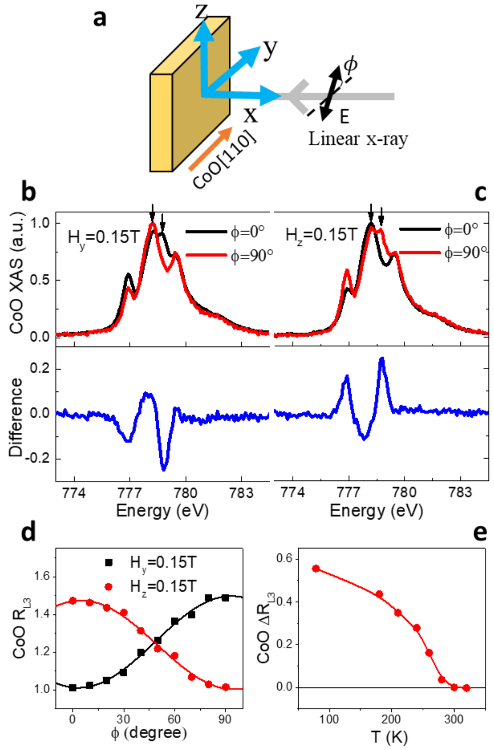

Supplementary Figure 2| XMLD of the AFM CoO layer within Py(30 nm)/Ag(2 nm)/CoO(2.5 nm)/MgO(001). a, Schematic drawing of XMLD measurements with linearly polarized x-rays at normal incidence. **b,c,** CoO L_3 edge x-ray absorption spectra (XAS) at the polarization angles of $\phi = 0°$ and $\phi = 90°$ for a 0.15 T magnetic field applied along the y-axis (H_y=0.15 T) and the z-axis (H_z=0.15 T), respectively. The difference of these two spectra shows the AFM CoO XMLD effect. **d** CoO L_3 ratio R_{L3} as a function of x-ray polarization angle at H_y=0.15 T and H_z=0.15 T, respectively. The opposite behavior observed for the two field orientations shows that the AFM CoO spins are coupled perpendicular to the Py spins. **e** CoO L_3 ratio difference ΔR_{L3} as a function of temperature. The disappearance of ΔR_{L3} at T=280K shows that the 2.5nm CoO has a Néel temperature of T_N=280K.

Supplementary Note 3. Separation of spin current- and interlayer coupling-driven $Fe_{75}Co_{25}$ spin procession

Considering the $Fe_{75}Co_{25}$ spin precession driven by an rf field, Py/$Fe_{75}Co_{25}$ interlayer magnetic coupling, and a spin current from the Py FMR, The corresponding Landau-Lifshifts-Gilbert equation is given by [2,3]

$$-\frac{d\vec{m}_{\rm FeCo}}{dt} = \gamma\vec{m}_{\rm FeCo}\times(\vec{H}_{\rm eff}+\vec{h}_{\rm rf}) - \alpha_{\rm FeCo}\vec{m}_{\rm FeCo}\times\frac{d\vec{m}_{\rm FeCo}}{dt} + \gamma\vec{m}_{\rm FeCo}\times J_{\rm int}\vec{m}_{\rm Py} + \alpha^{\rm sp}_{\rm Py}\vec{m}_{\rm Py}\times\frac{d\vec{m}_{\rm Py}}{dt} \qquad (1).$$

Here $\vec{m}_{\rm FeCo}$ and $\vec{m}_{\rm Py}$ are the $Fe_{75}Co_{25}$ and Py magnetization unit vectors, respectively, $\vec{H}_{\rm eff}$ is the effective field acting on the $Fe_{75}Co_{25}$ layer, including both the external field and the anisotropy field, $\vec{h}_{\rm rf}$ is the rf field from the CPW, $\alpha_{\rm FeCo}$ is the $Fe_{75}Co_{25}$ damping parameter, $-J_{\rm int}\vec{m}_{\rm Py}\cdot\vec{m}_{\rm FeCo}$ is the Py/$Fe_{75}Co_{25}$ magnetic interlayer coupling energy, and $\alpha^{\rm sp}_{\rm Py}$ is the coefficient for spin pumping from the Py FMR. We have ignored spin pumping from $Fe_{75}Co_{25}$ back to Py because of the much smaller amplitude of precession in the $Fe_{75}Co_{25}$ relative to that in the Py. The first two terms in Supplementary Equation (1) describe the $Fe_{75}Co_{25}$ spin precession without the influence of the Py. The last two terms in Supplementary Equation (1) describes additional spin excitation driven by the Py FMR by means of interlayer coupling and pumping of a spin current. The physical meaning of the spin current term is that conservation of angular momentum converts the extra damping of Py spin into a spin current injection into the neighboring layer. Adding the two driving torques [the last two terms in Supplementary Equation (1)] to the rf field driving toque and with the equilibrium magnetization along the z-axis, the total driving torque on the excitation of $\vec{m}_{\rm FeCo}$ in the spin precession plane (xy-plane) is

$$\vec{\tau} \approx \hat{z}\times\gamma\left(\vec{h}_{\rm rf}+J_{\rm int}\vec{m}_{\rm Py}\right) + \alpha^{\rm sp}_{\rm Py}\hat{z}\times\frac{d\vec{m}_{\rm Py}}{dt} = \hat{z}\times\gamma\left(\vec{h}_{\rm rf}+J_{\rm int}\vec{m}^{\rm AC}_{\rm Py}+\frac{\alpha^{\rm sp}_{\rm Py}}{\gamma}\omega\hat{z}\times\vec{m}^{\rm AC}_{\rm Py}\right)$$

where $\vec{m}^{\rm AC}_{\rm Py}$ is the precession component of the Py spin. Thus, the $Fe_{75}Co_{25}$ magnetization is actually driven by an equivalent total rf field of

$$\vec{h}^{\rm t}_{\rm rf} = \vec{h}_{\rm rf} + J_{\rm int}\vec{m}^{\rm AC}_{\rm Py} + \frac{\alpha^{\rm sp}_{\rm Py}}{\gamma}\omega\hat{z}\times\vec{m}^{\rm AC}_{\rm Py} \qquad (2).$$

In other words, the $Fe_{75}Co_{25}$ precession is driven by an effective rf field of $\vec{h}^{\rm t}_{\rm rf}$ after adding the interlayer coupling and spin current generated by the Py FMR. The significance of Supplementary Equation (2) is that, although both the interlayer-coupling term and the spin-current term lead to a peak in the the total rf driving field magnitude at the Py FMR point, the phases of the two terms differ by 90°, which in turn lead to a different phase of the $Fe_{75}Co_{25}$ spin precession. If the Py spin has precession amplitude $|\vec{m}^{\rm AC}_{\rm Py}| = \frac{\gamma h_{\rm rf}}{\alpha_{\rm Py}\omega}\sin\varphi_{\rm Py}$ ($\alpha_{\rm Py}$ is the Py damping coefficient) and phase $\varphi_{\rm Py}$ (defined as the phase angle of $\vec{m}^{\rm AC}_{\rm Py}$ relative to $\vec{h}_{\rm rf}$), and considering the Py and $Fe_{75}Co_{25}$ magnetization magnitudes ($M_{\rm Py}$and $M_{\rm FeCo}$) and thicknesses ($t_{\rm Py}$ and $t_{\rm FeCo}$), it is straightforward to derive from Supplementary Equation (2) that [4]:

$$\left|\frac{A_{\rm FeCo}}{A^0_{\rm FeCo}}\right| = \sqrt{1+\left(\beta^2_{\rm int}+\beta^2_{\rm sc}\right)\sin^2\varphi_{\rm Py}+2\beta_{\rm int}\sin\varphi_{\rm Py}\cos\varphi_{\rm Py}+2\beta_{\rm sc}\sin^2\varphi_{\rm Py}} \qquad (3).$$

$$\tan(\varphi_{\rm FeCo}-\varphi^0_{\rm FeCo}) = \frac{\beta_{\rm int}\sin^2\varphi_{\rm Py}-\beta_{\rm sc}\sin\varphi_{\rm Py}\cos\varphi_{\rm Py}}{1+\beta_{\rm int}\sin\varphi_{\rm Py}\cos\varphi_{\rm Py}+\beta_{\rm sc}\sin^2\varphi_{\rm Py}} \qquad (4).$$

Here $\beta_{\rm int} = \frac{M_{\rm Py}t_{\rm Py}}{M_{\rm FeCo}t_{\rm FeCo}}\cdot\frac{\gamma J_{\rm int}}{\alpha_{\rm Py}\omega}$ and $\beta_{\rm sc} = \frac{M_{\rm Py}t_{\rm Py}}{M_{\rm FeCo}t_{\rm FeCo}}\cdot\frac{\alpha^{\rm sp}_{\rm Py}}{\alpha_{\rm Py}}$ are the interlayer coupling and the spin current coefficients, which quantify the interlayer coupling contribution and the spin current contribution to the $Fe_{75}Co_{25}$ spin precession, respectively. $A^0_{\rm FeCo}$ and $\varphi^0_{\rm FeCo}$ are the $Fe_{75}Co_{25}$ spin precession amplitude and phase, respectively, driven by the CPW rf field only ($\vec{h}_{\rm rf}$). Therefore Supplementary Equation (3) and Supplementary Equation (4) describe the modification of the magnitude ($A_{\rm FeCo}$) and phase ($\varphi_{\rm FeCo}$) of the $Fe_{75}Co_{25}$ spin precession by the interlayer coupling effect ($\beta_{\rm int}$) and the spin current effect ($\beta_{\rm sc}$).

Supplementary Note 4. Interlayer coupling between Py and FeCo across Ag/CoO/Ag

Element-resolved hysteresis loops were measured using XMCD for the Py/Ag/CoO/Ag(2nm)/$Fe_{75}Co_{25}$ and Py/Ag/CoO/Ag(10nm)/$Fe_{75}Co_{25}$ samples. The Py/Ag/CoO/Ag(2nm)/$Fe_{75}Co_{25}$ sample has identical Py and $Fe_{75}Co_{25}$ coercivities [Supplementary Figure 3**a**], indicating the existence of Py/$Fe_{75}Co_{25}$ interlayer coupling across the Ag/CoO/Ag(2nm) spacer, so that switching one FM layer's magnetization causes the switching of the other FM layer's magnetization (there may be an error in the absolute coercivity value due to the residual magnetic field of the electromagnet). In contrast, the Py/Ag/CoO/Ag(10nm)/$Fe_{75}Co_{25}$ sample exhibits independent Py and $Fe_{75}Co_{25}$ coercivities [Supplementary Figure 3**b**], suggesting the absence of (or negiligible) Py/$Fe_{75}Co_{25}$ interlayer coupling across the Ag/CoO/Ag(10nm) spacer. This conclusion is further supported by comparing the FMR resonance fields of the Py/Ag/CoO/Ag/$Fe_{75}Co_{25}$ and Ag/CoO/Ag/$Fe_{75}Co_{25}$ samples. Note that the interlayer coupling $-J_{\rm int}\vec{\boldsymbol{m}}_{\rm Py}\cdot\vec{\boldsymbol{m}}_{\rm FeCo}$ is equivalent to a magnetic field of $H_{\rm int}=J_{\rm int}\vec{\boldsymbol{m}}_{\rm Py}$ applied to the $Fe_{75}Co_{25}$ magnetization. The dc part of $J_{\rm int}\vec{\boldsymbol{m}}_{\rm Py}^{\rm DC}$ reduces the $Fe_{75}Co_{25}$ FMR resonance field and the ac part of $J_{\rm int}\vec{\boldsymbol{m}}_{\rm Py}^{\rm AC}$ provides an additional driving rf field to the $Fe_{75}Co_{25}$ spin precession (discussed in the previous section). Indeed the $Fe_{75}Co_{25}$ FMR in Py/Ag/CoO/Ag(2nm)/$Fe_{75}Co_{25}$ shows a reduction of the FMR resonance field as compared to that in Ag/CoO/Ag(2nm)/$Fe_{75}Co_{25}$ [Supplementary Figure 3**c**], providing further evidence the for interlayer coupling between Py and $Fe_{75}Co_{25}$ across the Ag/CoO/Ag(2nm) spacer. In contrast, we observed identical $Fe_{75}Co_{25}$ FMR in Py/Ag/CoO/Ag(10nm)/$Fe_{75}Co_{25}$ and Ag/CoO/Ag(10nm)/$Fe_{75}Co_{25}$ samples [Supplementary Figure 3**d**], showing the absence (or negiligible) Py/$Fe_{75}Co_{25}$ interlayer coupling across the Ag/CoO/Ag(10nm) spacer. The FMR field difference with and without the Py layer at different temperatures (defined as H_1-H_2 in Supplementary Figure 3**e**) shows that the 10nm Ag layer decouples the Py and the $Fe_{75}Co_{25}$ layers, and that the sample with the 2nm Ag layer retains a temperature-independent Py/$Fe_{75}Co_{25}$ interlayer coupling, which is consistent with the temperature-independent $\beta_{\rm int}$ value in the main text [Fig. 2**f**]. While it was shown recently that AFM magnons can in certain cases mediate an interlayer coupling between two FM layers [5], the weak temperature dependence of the Py/$Fe_{75}Co_{25}$ interlayer coupling requires additional study.

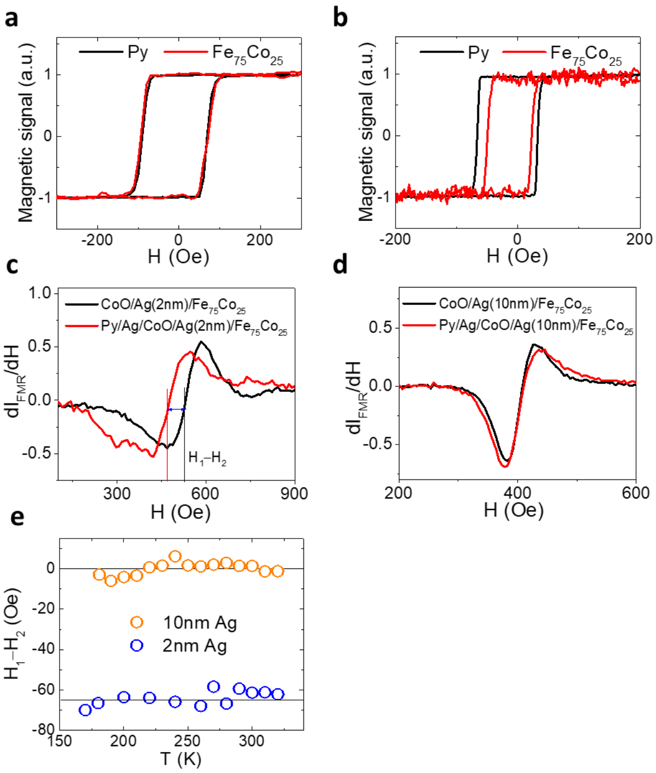

Supplementary Figure 3| Interlayer coupling between Py and $Fe_{75}Co_{25}$ layers across Ag/CoO/Ag. Hysteresis loops for the Py and $Fe_{75}Co_{25}$ layers obtained using XMCD at 200 K from **a** Py/Ag/CoO/Ag(2nm)/$Fe_{75}Co_{25}$ and **b** Py/Ag/CoO/Ag(10nm)/$Fe_{75}Co_{25}$ samples. FMR spectra of the $Fe_{75}Co_{25}$ layer with (red line) and without (black line) the Py layer measured at 14 GHz for the **c** d_{Ag}=2 nm and **d** d_{Ag}=10 nm samples. **e** Shift of $Fe_{75}Co_{25}$ layer resonance field due to the Py capping as a function of temperature for the two samples.

Regarding nature of the coupling between a FM layer and the CoO through a Ag layer, there could be RKKY, pinhole mediation, or dipolar couplings. Our previous work [6] shows that the interlayer coupling between Fe and CoO through Ag oscillates with the Ag thickness with a peak at 2nm Ag and decays to a negligible value for Ag thicker than ~5nm. The oscillatory behavior is a signature of RKKY interaction rather than pinhole mediation or dipolar couplings. In addition, the existence of oscillatory coupling also indicates that the flatness of the Ag spacer layer is better than a fraction of a nanometer. In fact, our previous x-ray pump-probe measurements of coupling through an insulating MgO layer [3] show that the sample quality is such that there is negligible pinhole and dipolar coupling. Finally, any coupling term should produce a monopolar phase behavior, regardless of the origin of the coupling. Therefore we conclude that the results obtained from the Py/Ag/CoO/Ag(10nm)/$Fe_{75}Co_{25}$ sample, particularly the bipolar phase behavior, are due to transmission of a coherent ac spin current through the CoO.

Supplementary Note 5. Uncompensated FM Co spins in CoO

In order to make sure that the observed ac XMCD signal at the Co edge originates solely from the $Fe_{75}Co_{25}$ layer, we carried out additional ac XMCD measurements of the CoO layer at the Co L_3 edge [Supplementary Figure 4**b**]. It is well known that oxygen migration at the interface between CoO and a metal layer [7] (e.g. Ag in our case) or the magnetic interaction between the FM and AFM layers across the NM spacer layer [8] can result in a fraction of a monolayer of uncompensated ferromagnetic Co spins at the interface. XMCD and XMLD measurements at the Co edge were used to identify potentially uncompensated FM moments and the expected compensated AFM spins in the CoO layer. DC XMCD measurements at the Co edge in Py(30nm)/Ag(2nm)/CoO(2.5nm)/MgO(001) indeed yield a small XMCD signal, equivalent to ~0.066 nm FM metallic Co layer when compared to the XMCD signal from a 1.25 nm metallic Co layer [Supplementary Figure 4**c**]. After inserting a thin NiO(0.9nm) layer between the Ag and CoO, the Co XMCD signal disappeared from static measurements [blue lines in Supplementary Figure 4**c**,**d**], showing that the uncompensated FM Co spins in CoO emerge at the Ag/CoO interface.

Ac XMCD measurements of the uncompensated FM Co spins were carried out at 4GHz, but did not yield a measurable signal. However, by adjusting the microwave frequency to 2 GHz, we were able to increase the power output of the frequency generator and deliver more rf power to the sample. However, in this configuration we were limited to exciting the Py FMR at 280K with a resonance field of 80 Oe. We performed ac XMCD measurements on Py(30nm)/Ag(2nm)/CoO(2.5nm)/MgO(001) at 20 Oe, 80 Oe, and 150 Oe. Supplementary Figure 4**a**,**b** clearly shows that the uncompensated FM Co spins precess only near the Py FMR field (80 Oe). The Co ac XMCD signal in the Py/Ag/NiO/CoO sample is greatly suppressed [Supplementary Figure 4**e**], indicating that most of the Co ac XMCD signal in Py/Ag/CoO originates from an induced spin precession of the uncompensated Co spins at the Ag/CoO surface. The absence of Py FMR at low temperatures (less than ~270K) at 2GHz makes it impossible to make temperature-dependent and field-dependent measurements on the uncompensated FM Co spins. Therefore, we grew a Py(30nm)/Ag(2nm)/Co(1nm)CoO(2.5nm)/MgO(001) sample to simulate the behavior of the FM Co spins at the Ag/CoO interface and performed ac XMCD measurements at 4 GHz (main text). We note that even with the greater microwave power used at 2GHz, the uncompensated FM Co ac XMCD is only a small fraction of the Co ac XMCD signal at 4GHz in Py/Ag/CoO/Ag(10nm)/$Fe_{75}Co_{25}$ (at least less than 20%) This is strong evidence that the Co ac XMCD signal at 4GHz in the Py/Ag/CoO/Ag/$Fe_{75}Co_{25}$ system originates mostly from the $Fe_{75}Co_{25}$ layer.

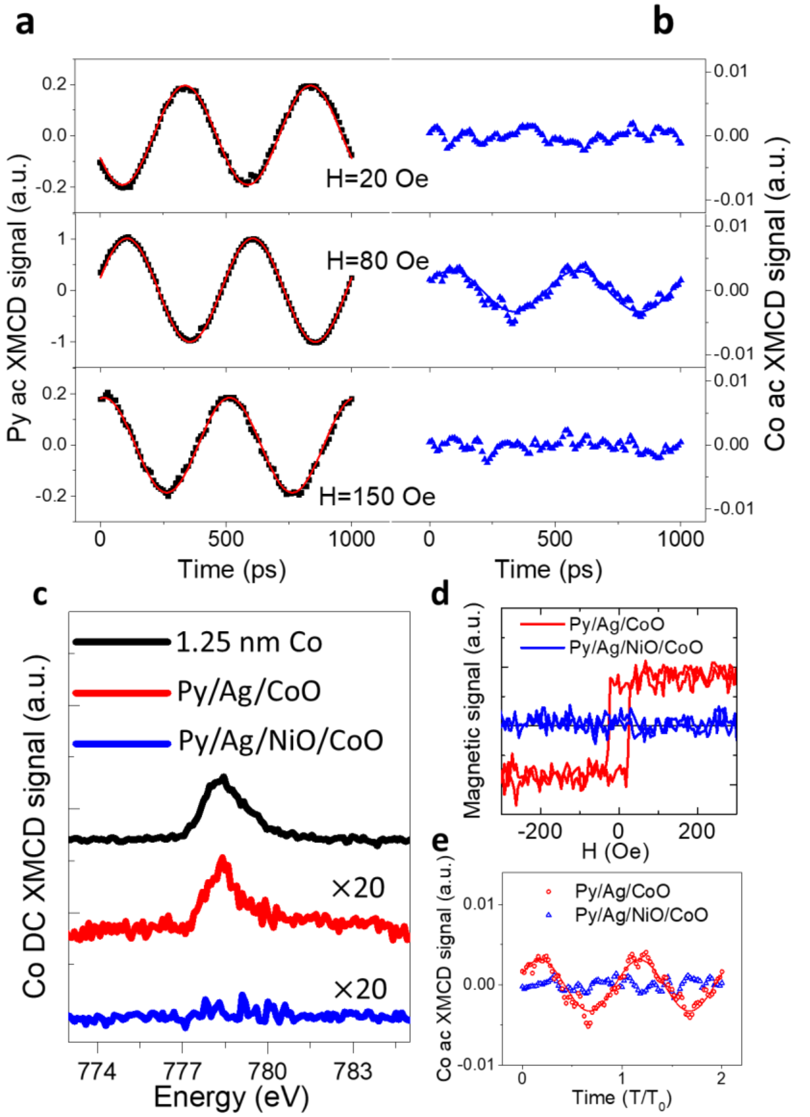

Supplementary Figure 4| Uncompensated FM Co at the Ag/CoO interface in Py(30nm)/Ag(2nm)/CoO(2.5nm)/MgO(001). a Py and **b** uncompensated FM Co ac XMCD signals at 2 GHz and T=280K at fields around the Py FMR resonance field of 80 Oe. **c** DC XMCD signals at the Co L_3 edge obtained from metallic Co(1.25nm), Py(30nm)/Ag(2nm)/CoO(2.5nm), and Py(30nm)/Ag(2nm)/NiO(0.9nm)/CoO(2.5nm). **d** Hysteresis loops of Co XMCD signal with and without the NiO layer. **e** Co ac XMCD signal with and without the NiO layer.

References:

1. Wu, J. et al. Direct measurement of rotatable and frozen CoO spins in exchange bias system of CoO/Fe/Ag(001). *Phys. Rev. Lett.* **104**, 217204 (2010).
2. Baker, A. A. et al. Anisotropic absorption of pure spin currents. *Phys. Rev. Lett.* **116**, 047201 (2016).
3. Li, J. et al. Direct detection of pure ac spin current by x-ray pump-probe measurements. *Phys. Rev. Lett.* **117**, 076602 (2016).
4. Ghosh, A., Auffret, S., Ebels, U. & Bailey, W. E. Penetration depth of transverse spin current in ultrathin ferromagnets. *Phys. Rev. Lett.* **109**, 127202 (2012).
5 . Cheng, R., Xiao, D. & Zhu, J. G. Interlayer Couplings Mediated by Antiferromagnetic Magnons. *Phys. Rev. Lett.* **121**, 207202 (2018).
6. Meng, Y. et al. Magnetic interlayer coupling between antiferromagnetic CoO and ferromagnetic Fe across a Ag spacer layer in epitaxially grown CoO/Ag/Fe/Ag(001). *Phys. Rev. B* **85**, 014425 (2012).
7. Gilbert, D. A. et al. Controllable positive exchange bias via redox-driven oxygen migration. *Nat. Commun.* **7**, 11050 (2016).
8. Valev, V. K., Gruyters, M., Kirilyuk, A. & Rasing, Th. Direct observation of exchange bias related uncompensated spins at the CoO/Cu Interface. *Phys. Rev. Lett.* **96**, 067206 (2006).
